# Matured Myofibers in Bioprinted Constructs with In Vivo Vascularization and Innervation

**DOI:** 10.3390/gels7040171

**Published:** 2021-10-15

**Authors:** Catherine G. Y. Ngan, Anita Quigley, Richard J. Williams, Cathal D. O’Connell, Romane Blanchard, Mitchell Boyd-Moss, Tim D. Aumann, Penny McKelvie, Gordon G. Wallace, Peter F. M. Choong, Rob M. I. Kapsa

**Affiliations:** 1 Department of Surgery, The University of Melbourne, St Vincent’s Hospital, Melbourne, VIC 3010, Australia; romane.blanchard@gmail.com (R.B.); pfchoong@gmail.com (P.F.M.C.); 2 Department of Orthopedics, St Vincent’s Hospital, Melbourne, VIC 3010, Australia; 3 ARC Centre of Excellence for Electro Materials Science, University of Melbourne, Melbourne, VIC 3010, Australia; anita.quigley@rmit.edu.au (A.Q.); gwallace@uow.edu.au (G.G.W.); 4 Biofab3D@ACMD, St Vincent’s Hospital, Melbourne, VIC 3010, Australia; richard.williams@deakin.edu.au (R.J.W.);; 5 Department of Clinical Neurosciences, St Vincent’s Hospital, Melbourne, VIC 3010, Australia; 6 Department of Medicine, The University of Melbourne, Melbourne, VIC 3010, Australia; 7 School of Engineering, RMIT University, Bundoora, VIC 3000, Australia; mboydmoss@gmail.com; 8 School of Medicine, Deakin University, Waurn Ponds, VIC 3010, Australia; 9 Florey Institute of Neuroscience and Mental Health, Parkville, VIC 3052, Australia; timothy.aumann@florey.edu.au; 10 Department of Anatomical Pathology, St Vincent’s Hospital, Melbourne, VIC 3010, Australia; penny.mckelvie@svha.org.au; 11 ARC Centre of Excellence for Electro materials Science, University of Wollongong, Wollongong, NSW 2522, Australia

**Keywords:** skeletal muscle, tissue engineering, neuromuscular, bioprinting, GelMA

## Abstract

For decades, the study of tissue-engineered skeletal muscle has been driven by a clinical need to treat neuromuscular diseases and volumetric muscle loss. The in vitro fabrication of muscle offers the opportunity to test drug-and cell-based therapies, to study disease processes, and to perhaps, one day, serve as a muscle graft for reconstructive surgery. This study developed a biofabrication technique to engineer muscle for research and clinical applications. A bioprinting protocol was established to deliver primary mouse myoblasts in a gelatin methacryloyl (GelMA) bioink, which was implanted in an in vivo chamber in a nude rat model. For the first time, this work demonstrated the phenomenon of myoblast migration through the bioprinted GelMA scaffold with cells spontaneously forming fibers on the surface of the material. This enabled advanced maturation and facilitated the connection between incoming vessels and nerve axons in vivo without the hindrance of a scaffold material. Immunohistochemistry revealed the hallmarks of tissue maturity with sarcomeric striations and peripherally placed nuclei in the organized bundles of muscle fibers. Such engineered muscle autografts could, with further structural development, eventually be used for surgical reconstructive purposes while the methodology presented here specifically has wide applications for in vitro and in vivo neuromuscular function and disease modelling.

## 1. Introduction

Skeletal muscle is a dynamic, vascularized, and innervated tissue that supports stature and all voluntary movement in the body. While it has the capacity to regenerate and self-repair from small injuries, volumetric muscle defects and genetic myopathies contribute to a significant healthcare burden [1,2]. The study of these diseases and the development of their treatments require faithful models of skeletal muscle physiology and anatomy in order to demonstrate efficacy and safety prior to translation into clinical trials [3,4]. However, the search for muscle models in vitro has been limited by biofabrication techniques that result in poor diffusion of cell nutrients, are not permissive for the integration of nerves and vessels, and, as a consequence, hinder maturation of the engineered tissues [5].

The neogenesis of skeletal muscle in vitro relies on the emulation of the in vivo environment, which must simulate the regenerative cell niche to sufficiently direct and sustain the differentiation of muscle progenitors from regenerative myoblasts to functional multinucleated myofibers. While traditional muscle regeneration methods endeavor to recapitulate the stem cell niche in two-dimensional cultures, such techniques do not translate well to the fabrication of larger constructs for clinical applications [5]. Three-dimensional (3D) cultures introduce volume, can better promote cell maturation, and offer more accurate models of cell interaction with other systems such as nerves and vasculature [6,7,8].

The key to the 3D engineering of skeletal muscle is the fabrication of a porous scaffold to allow for the diffusion of nutrients and cell waste while mimicking the native mechanical and biochemical properties of muscle. Bioprinting, a fabrication technique that involves the extrusion of cell-carrying hydrogel bioinks into multilayered filaments, has the potential to address these conditions. This would require the development of a muscle-specific ink, which could form the scaffold for successful cell culture while possessing the material properties required for printing [9,10]. A candidate for this ink is gelatin methacryloyl (GelMA), one of the few biomaterials that can print free-standing structures without a secondary support material due to its gelation properties at low temperatures [11,12,13]. Its tunable mechanical and biochemical properties make it a versatile material for tissue engineering, and early work with immortalized myoblast cell lines has demonstrated cell attachment and high cell viability [14,15,16,17,18].

Following the careful in vitro construction of fabricated muscle, survival in vivo depends on the rapid development of critical neuromuscular connections and neovasculature. Common sites of implantation, such as the subcutaneous space or adjacent-to-muscle compartments, are poor sources of blood vessels, which would significantly limit the development of muscle and its neural connection [9,10,19]. An alternative system is to use a surgically created arteriovenous loop (AV loop) in a dedicated tissue chamber. This approach creates a vascular pedicle for the tissue that can be harvested wholly for transplantation elsewhere [20,21,22,23]. For skeletal muscle, the further inclusion of a motor nerve, along with the AV loop, would provide an optimal environment for the innervation and vascularization of the tissue-engineered construct [24]. The use of an in vivo culture chamber isolates the system for the interrogation of specific interactions between the neurovascular structures and the muscle construct as well as minimizing any accidental harvest of native muscle.

Much of the existing literature has focused on the mechanical and chemical characterizations of potential scaffold biomaterials with subsequent examination of myoblasts limited to a perfunctory demonstration of in vitro cell viability and the alignment of myotubes. In contrast, this study investigated GelMA as a bioink specifically for skeletal muscle tissue engineering with systematic assessment of cytotoxic elements in the bioprinting process to optimize the material for in vitro myogenesis. Finally, the bioprinted muscle structures were implanted in an AV loop chamber, to assess their viability and development in vivo and to interrogate their potential for application in regenerative medicine.

## 2. Results

### 2.1. Optimizing Myoblast Culture in Cast GelMA Samples

**Figure 1:**
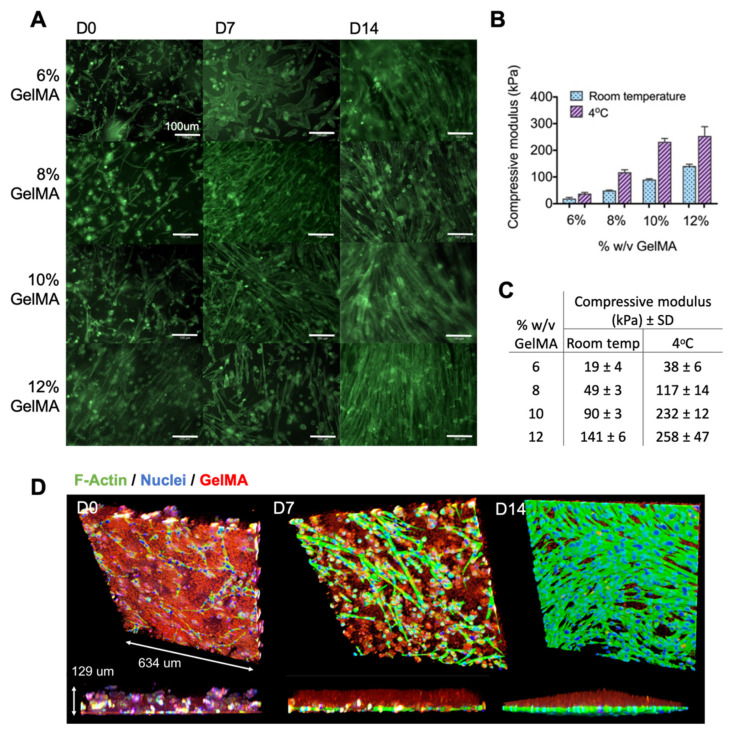
Characterization of GelMA concentrations. (**A**) Myoblasts were encapsulated in 6%, 8%, 10%, and 12% *w*/*v* GelMA and differentiated over 14 days to establish the optimal formulation for myo-regenerative cells. Samples were fixed and stained for F-actin at days 0, 7, and 14, revealing similar myotube morphology in all concentrations. (**B**,**C**) Compressive moduli of the different percentages of GelMA samples at room temperature and at 4 °C, measured without cells. Error bars represent standard deviation. (**D**) The 3D rendered confocal images of myoblasts encapsulated in GelMA, with images taken at days 0, 7, and 14 of differentiation. A total of 8% *w*/*v* GelMA was chosen as a representative sample. Myofibers were stained for F-actin (green) and DNA (blue), and GelMA was demarcated with red fluorescent latex beads. These images demonstrate the migration of myoblasts to the boundary of the material, where they subsequently differentiated into multinuclear myotubes.

**Figure A1:**
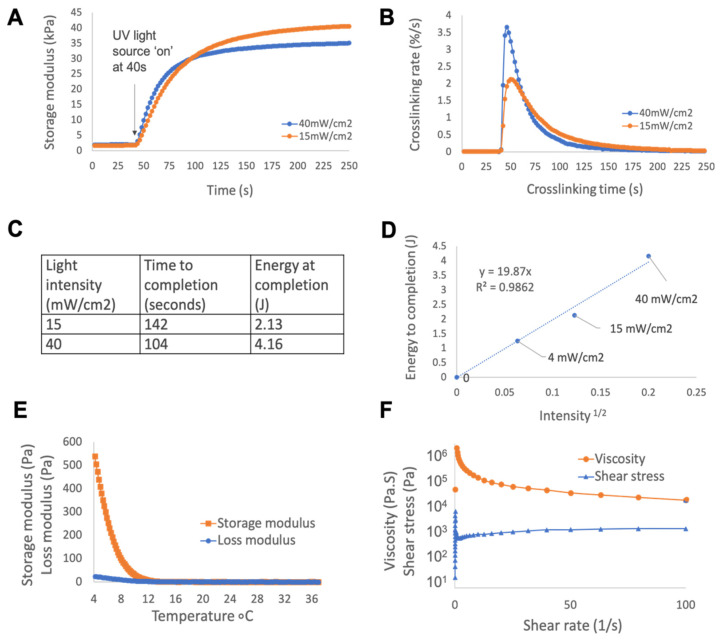
Rheology data for 8% *w*/*v* GelMA/0.1% *w*/*v* LAP, performed at 4 °C unless otherwise specified. (**A**) To calculate crosslinking times of GelMA at 4 mW/cm^2^, the material was more rapidly crosslinked at 40 mW/cm^2^ and 15 mW/cm^2^ with the purpose of extrapolating the data to 4 mW/cm^2^. This was done to minimize the effect of condensation between the material and stage (a consequence of using a quartz plate to allow for in situ UV curing). (**B**) The relative reaction rates for these intensities were defined as the change in storage modulus per second as a percentage of the final modulus after 210 s of curing time, a timepoint chosen to approximate completion as it occurs well within the range of the graph approaching plateau. Energy to completion was defined as a relative reaction rate of <0.1%/s, which occurred at 142 s at 15 mW/cm^2^, and 104 s at 40 mW/cm^2^. (**C**) The energy to crosslink the material could then be estimated by multiplying time (t) by intensity (I): E=tI. (**D**) Known relationships between light intensity and crosslinking rate were used to extrapolate this data: $E=k_{E}\sqrt{\frac{I}{c}}$, where E is the total energy, $k_{E}$ is the proportionality coefficient, I is the intensity of light, and c is the concentration of photoinitiator [47]. These energy values were plotted against $\sqrt{I}$ to form a line of best fit through the origin. From this linear relationship, the energy required to crosslink GelMA at 4 mW/cm^2^ was 1.26 J. By applying E=tI, the minimum time thus required to crosslink GelMA at the desired 4 mW/cm^2^ was 314 s. To establish a safe margin ensuring the material was fully crosslinked, this timeframe was rounded up to 400 s and used for all subsequent UV-curing experiments at 4 mW/cm^2^. (**E**) Temperature analysis of non-crosslinked GelMA from 37 °C to 4 °C demonstrated a sol-gel transition at 15 °C. (**F**) The viscosity was then immediately assessed after cooling to reflect bioprinting conditions. The material exhibited typical shear-thinning properties at 4 °C.

**Figure A2:**
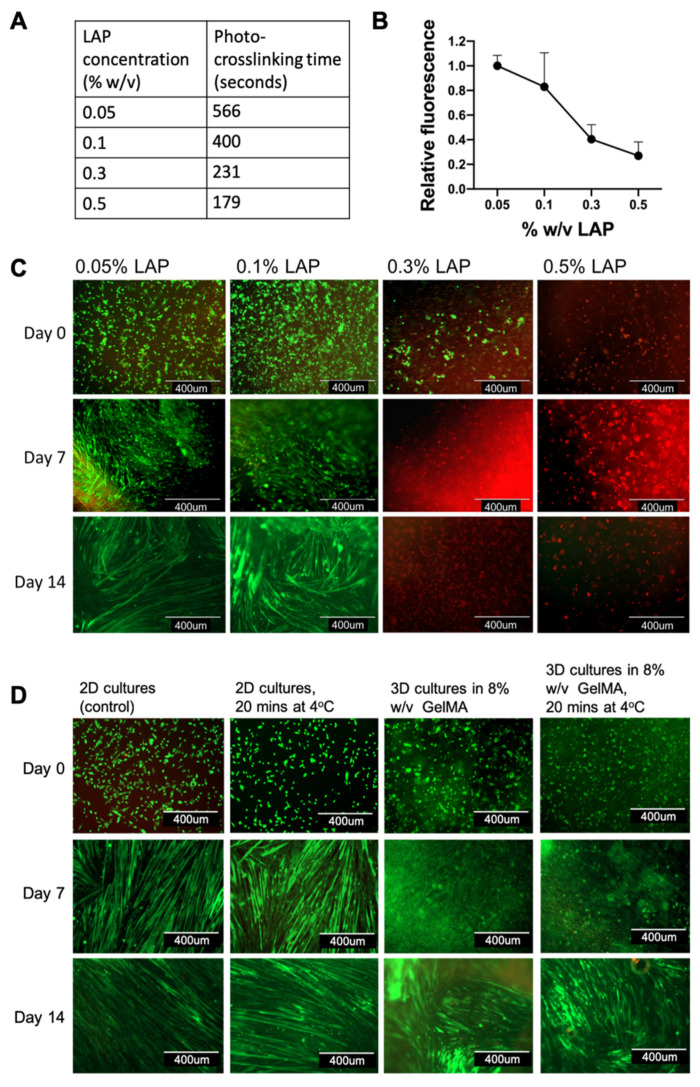
Optimization of photocuring conditions for myoblast viability in GelMA. (**A**) A range of UV crosslinking times were calculated for different concentrations of lithium phenyl-2,4,6-trimethylbenzoylphosphinate (LAP), based on known kinetic relationships for GelMA between energy, light intensity, and crosslinking rate. This was to achieve the same degree of crosslinking between different photocuring configurations. A very low intensity of UV light (4 mW/cm^2^) was chosen to minimise cytotoxicity. (**B**) These conditions were applied to myoblasts encapsulated in 8% *w*/*v* GelMA in a series of cytotoxicity screening tests. Metabolic assays (CellTitre-Blue) of cells in proliferation demonstrated a clear threshold of toxicity associated with higher concentrations of LAP. Error bars represent standard deviation. (**C**) Live (green) and dead (red) cell stains over two weeks of differentiation similarly demonstrated high cell viability in cultures with ≤0.1% *w*/*v* LAP, while cultures with ≥0.3% *w*/*v* LAP had no viable cells by the end of the study. In addition, the myoblasts in 0.05% and 0.1% LAP were able to mature into myofibers. (**D**) A further cell viability study was conducted with the additional condition of cooling cell cultures to 4 °C. Myoblasts grown on tissue culture plastic (2D cultures) and encapsulated in 8% *w*/*v* GelMA (3D cultures) were stored at 4 °C for 20 min. The 3D cultures were then UV-crosslinked, and all cultures were incubated in tissue culture conditions at 37 °C and 5% CO_2_. Subsequent live/dead cell stains showed that cooling to 4 °C did not adversely affect cell viability nor the ability for myoblasts to fuse into myofibers.

Myoblasts were encapsulated and photo-crosslinked in GelMA that had undergone rheological characterization and was optimized for cell viability, thus defining the LAP concentration, the temperature, and the photo-curing time (Appendix A Figure A1 and Figure A2). A similar degree of cell fusion and myotube formation was observed in all the GelMA concentrations, despite the compressive moduli ranging from 40 to 260 kPa (Figure 1). The 3D-rendered confocal micrographs demonstrated the cell migration through the GelMA over two weeks of culture. By day 14, most cells had migrated to the material boundary to form an extensive layer of myotubes. This experiment was performed in low-adhesion tissue culture plates to eliminate the possibility of cells growing on the plastic below the material. A total of 8% *w*/*v* GelMA was used for the subsequent experiments, given that there was no morphological difference between the GelMA concentrations.

### 2.2. Printing Myoblasts Encapsulated in a GelMA Bioink

**Figure 2:**
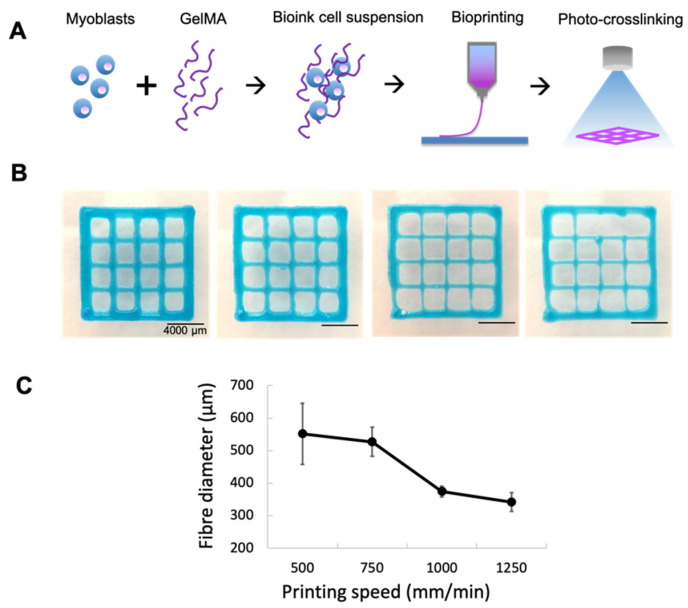
Parametric study of fiber diameters. Constructs were printed with the smallest available CELLINK conical nozzle (27 G) at the minimum pressure required to initiate and maintain bioink flow (60 kPa). Blue food dye was used for the purpose of imaging. (**A**) Schematic of printing process. (**B**) Printing speeds were compared between 500, 750, 1000, and 1250 mm/min (left to right); 1000 mm/min produced the finest fibers without thread break-up. (**C**) Fibers were soaked in PBS overnight at 37 °C before diameters were measured. Error bars represent standard deviation.

**Figure 3:**
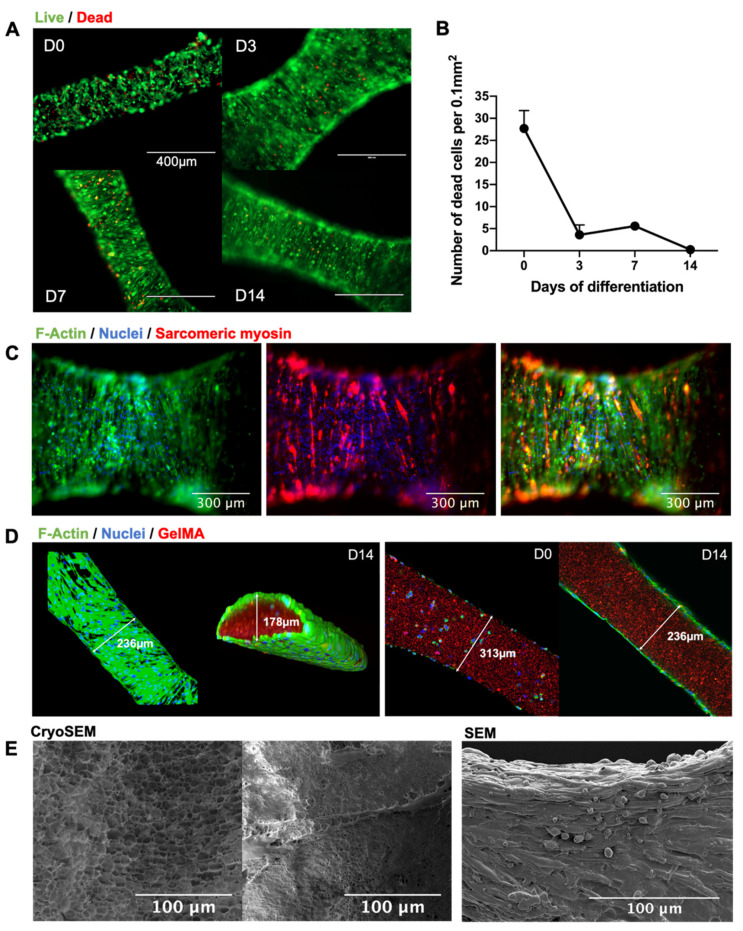
Characterizing myoblast behavior in bioprinted fibers. (**A**) Live (green) and dead (red) cell stains were performed at days 0, 3, 7, and 14 of differentiation after bioprinting. Given the nature of myoblast fusion, live cells were not individually counted, although, qualitatively, the live stain revealed dense myofiber formation. (**B**) Cell death was reported as a cell count per 0.1 mm^2^ of printed fiber. There was a peak in cell death at day 0, as would be expected due to shear stress during extrusion, followed by a rapid recovery from day 3 to day 14. Error bars represent standard deviation. (**C**) Cells were stained for F-actin (green), sarcomeric myosin (red), and DNA (blue). The emergence of myosin-positive fibers is a marker of maturing contractile mechanisms within the myofiber. (**D**) Confocal imaging of cells demonstrated migration from within the GelMA to the boundary of the material, where they fused into multinucleated myotubes on the material surface. (**E**) CryoSEM of bioprinted GelMA showed the nature of myofiber adherence without surface microgrooving or patterning that might have influenced the direction of cell growth. SEM demonstrated a dense multilayered myotube culture on the printed GelMA fibers.

The printing parameters were defined to produce the finest fibers without thread breakage with an average fiber diameter of 360 µm (Figure 2). Having determined the optimal printing speed, cell-laden GelMA (20 million cells/mL in 8% GelMA/0.1% LAP) was printed and photocured in a crosshatch pattern. The live and dead cell stains of the bioprinted fibers demonstrated high cell viability both immediately after printing and over two weeks of in vitro differentiation (Figure 3). Cells were again observed to migrate to the perimeters of the printed fibers, where they fused into myotubes on the GelMA surface. This was consistent with myoblast behavior in cast GelMA samples, along with the added observation that myoblasts could migrate out in all directions in the thinner bioprinted constructs. Imaging with cryoSEM further demonstrated an absence of microgrooves on the material surface that might have influenced the direction of the myofiber growth. The SEM permitted better preservation of cells on the material, and these images demonstrated multi-layered myotubes growing on the printed GelMA.

### 2.3. Gene Expression Analysis of Bioprinted Muscle

Five gene markers of myogenic maturation were analyzed: MYOG, MYF6, SIX4, MYH1, and MYH8. GAPDH was used as the housekeeping gene. MYOG and MYF6 represent two of the four key myogenic regulatory factors that control cell fusion and terminal differentiation, while SIX4 is one of the earliest regulators of myogenic lineage specification [25]. MYH1 and MYH8 encode for the key contractile protein myosin, which exists in several isoforms during development [26].

**Figure 4:**
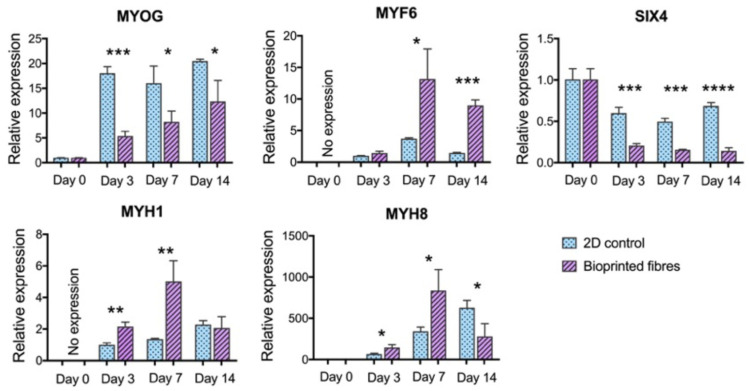
Gene expression demonstrated more advanced myogenesis in bioprinted fibers. The graphs represent the fold changes calculated with the 2^ΔΔCΤ^ method. There was a steady-state expression of MYOG in the 2D cultures after day 3, with significantly lower expression in the bioprinted samples at day 3 (*p* = 0.0002), day 7 (*p* = 0.0295), and day 14 (*p* = 0.0286). The increase in MYOG in bioprinted samples across timepoints was not statistically significant. MYF6 was undetectable at day 0, but over three-fold higher than the 2D control on day 7 (*p* = 0.0258) and day 14 (*p* = 0.0001). SIX4 was significantly downregulated in the bioprinted fibers compared to 2D controls at day 3 (*p* = 0.0008), day 7 (*p* = 0.0001), and day 14 (*p* < 0.0001). Upregulation of MYH8 was significantly greater in bioprinted fibers at day 3 (*p* = 0.0165), day 7 (*p* = 0.0292), and day 14 (*p* = 0.0279). Upregulation of MYH1 was significantly greater in the bioprinted fibers at day 3 (*p* = 0.0031) and day 7 (*p* = 0.0084). All asterisks refer to statistically significant differences between 2D control and bioprinted fibers at the same timepoint (* *p* < 0.05, ** *p* < 0.01, *** *p* < 0.001, **** *p* < 0.0001). Error bars represent standard deviation.

This analysis of myogenic markers revealed statistically significant differences in the gene expression between the 2D control and the bioprinted fibers, demonstrating an overall more mature phenotype in the bioprinted fibers (Figure 4). The joint upregulation of MYF6 and the downregulation of MYOG is consistent with advanced differentiation. MYOG is known to be expressed prior to terminal differentiation and MYF6 is expressed after myoblast fusion and its presence furthermore downregulates MYOG [27]. SIX4 was significantly downregulated in the bioprinted fibers as expected in more differentiated myofibers [28]. Both myosin isoforms were greatly upregulated at day 7 in bioprinted fibers with an overall trend of more accelerated myosin protein development when compared to the gradual increase in the 2D controls. The subsequent fall in gene expression at day 14 may represent a plateau in myofiber maturation for the bioprinted fibers under these conditions, while the 2D cultures did not reach the same level of myosin gene expression over two weeks.

### 2.4. Functional Analysis of Bioprinted Muscle

Differentiating myoblasts show spontaneous calcium transients due to immature mechanisms that allow flux from intracellular calcium stores and, later on, an influx of extracellular calcium through the immature expression of the T-type calcium channels, which in turn allow a voltage drift towards the depolarization threshold [29,30,31]. The latter mechanism can trigger regular pacemaker activity reminiscent of cardiac myocytes, which is a known feature of developing myofibers before they mature and reach electrical independence [31].

**Figure 5:**
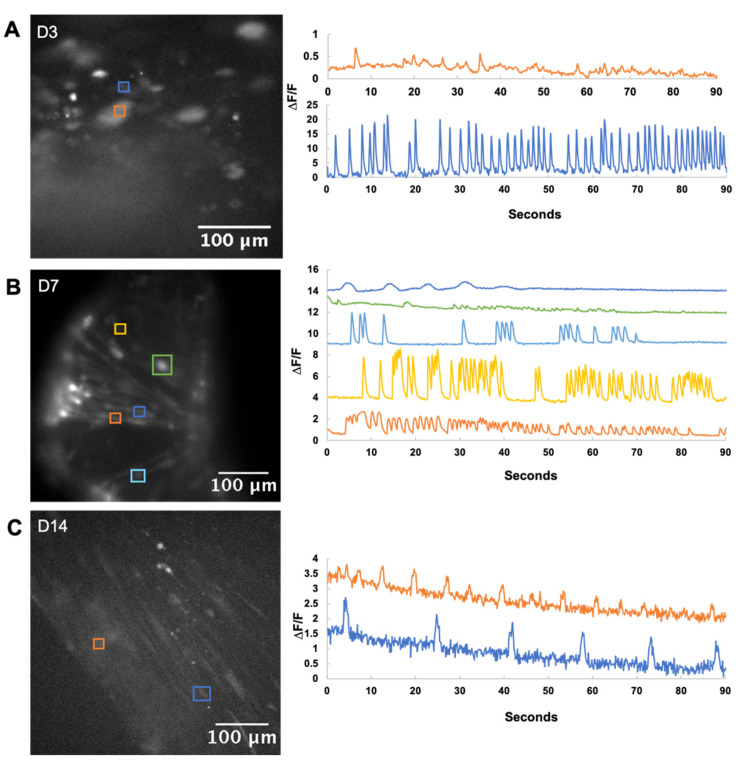
Calcium imaging of bioprinted fibers. Active cells are marked out in the micrographs on the left, with the calcium trace in the corresponding color on the right. (**A**) Day 3 bioprinted fibers showed minimal activity with rapid firing occasionally observed from single myoblasts. (**B**) Day 7 bioprinted fibers showed a variety of calcium activity, which is commonly observed during myoblast differentiation. (**C**) By Day 14, active cells had pacemaker activity, characterized by a slower, regular spiking pattern. This is thought to represent a maturation of calcium-handling proteins that can respond to depolarization as well as replenish intracellular stores in time for the next action potential.

As such, calcium imaging was used to characterize functionality in developing myotubes at days 3, 7, and 14 of differentiation (Figure 5). The amplitude of signal recordings was not quantitatively assessed due to the 3D nature of the culture which influences signal intensity, but the frequency and the pattern of activity were recorded. Widespread patterns of calcium flux were observed by day 7, reflective of the varying stages of maturation of the myofibers at this timepoint. By day 14, sustained regular pacemaker-like activity was observed in the developing myofibers. Fewer active cells were recorded, perhaps due to the maturing membrane channels that were less susceptible to the calcium flux.

### 2.5. Implantation of Bioprinted Skeletal Muscle Fibers In Vivo

**Figure 6:**
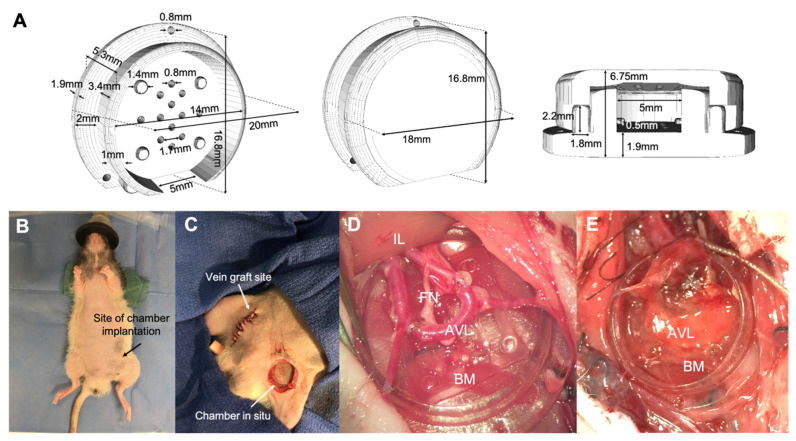
Bioprinted constructs were implanted in vivo for two weeks in an isolated chamber that housed an AV loop and transected femoral nerve. (**A**) The 3D-printed chambers comprised of a base on which the bioprinted grid was positioned, along with a lid that held the contents in place. An opening in the chamber wall allowed for entry and exit of the neurovascular bundle. Perforations in the base allowed for flow of cell media while in tissue culture conditions. (**B**,**C**) Chambers were positioned in a subcutaneous pocket in the left groin of the rat. Vein grafts were harvested from the right femoral vein to create the AV loop. (**D**) The AV loop and transected femoral nerve was placed on top of the bioprinted muscle grid. BM = bioprinted muscle, AVL = arteriovenous loop, FN = femoral nerve, IL = ilioinguinal ligament. (**E**) Muscle construct two weeks after in vivo implantation.

After one week of in vitro differentiation, the bioprinted muscle constructs were housed in the 3D-printed chambers supplied with a surgically formed AV loop and a transected femoral nerve in the nude rat model (Figure 6). The chamber was implanted and secured in a subcutaneous pocket in the groin for a further two weeks, after which the contents were retrieved for fixing and staining. The native muscle was not incised during this procedure.

**Figure 7:**
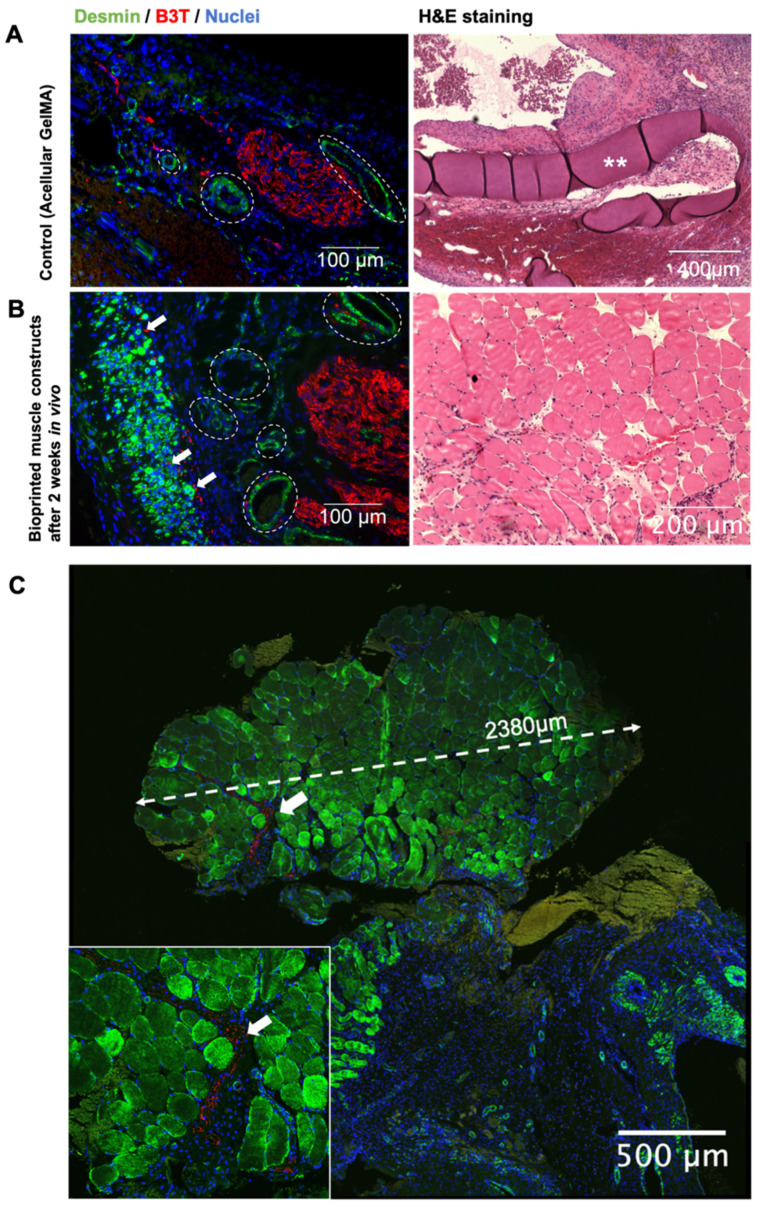
Immunostaining was performed on tissues harvested from the AV loop chambers after two weeks in vivo. Sections were stained for desmin (green), B3T (red), and DNA (blue). Examples of angiogenesis are marked with dotted lines. Arrows point to neuronal outgrowth. Asterisks label GelMA. (**A**) After two weeks of implantation, sections from the GelMA-only controls demonstrated angiogenesis and neuronal sprouting without muscle formation. Blood vessels were identified as ringed desmin-positive structures due to the smooth muscle encircling the vascular wall. Neuronal outgrowth was observed as clusters of B3T-positive cells. H&E staining revealed the inert characteristics of GelMA, which after two weeks showed no muscular, neural, or vascular growth within the material. (**B**) In contrast, tissue from chambers that housed bioprinted grids demonstrated muscle regeneration and maturation. Axonal outgrowth and angiogenesis were present throughout the developing muscle fibers. (**C**) Confocal micrograph of a 2.38 mm bundle of mature skeletal muscle from one of the chambers that housed bioprinted muscle. Inset is the magnified image of neuronal growth within this muscle bundle.

The chamber specimens were sectioned and stained for the muscle-specific protein desmin and the neuronal microtubule beta-III tubulin (Figure 7). Triads of muscle, neovasculature, and neuronal sprouting were observed. The regenerating multinucleated myofibers were identified by their centrally located nuclei, and early evidence of innervation was seen in the muscle bundles interspersed with the B3T-positive structures. Mature muscle morphology was also observed, characterized by sarcomeric striations and peripherally placed nuclei in organized bundles of parallel fibers. The new muscle tissue was largely independent of the original GelMA scaffold, and there was no integration of the vasculature or the nerves into the GelMA scaffold. Significant neural ingrowth was present in this bundle of mature muscle.

## 3. Discussion

This study presents a simple single-material bioprinting technique to engineer functional skeletal muscle fibers capable of advanced maturation for in vivo engineered muscle modelling. Through the systematic evaluation and optimization of GelMA as a bioink for myoblast cultures, new observations were made regarding cell maturation and migration in this biomaterial. Advanced myofiber development was observed by both molecular and functional analysis. After only two weeks of implantation in vivo, the engineered tissue was capable of vascular and neural integration. These results present a promising approach for rapid 3D fabrication of functional skeletal muscle constructs, with opportunities arising for both lab-based personalized neuromuscular tissue modelling and, ultimately, even for clinical applications.

In the process of optimizing the material for myoblast cultures, a key outcome was to recognize and subsequently capitalize on the phenomenon of cell migration. The 3D-rendered confocal imaging revealed the relocation of the myoblasts to the boundary of the material in both the cast and bioprinted samples. One explanation for this could be the presence of a nutrient diffusion gradient within the hydrogel matrix, as previously described by Wang et al. [32]. Nonetheless, despite the myoblasts rather resourcefully transforming a 3D-culture system into essentially a pseudo-2D one, this phenomenon was not at all a limitation in the differentiation process. Indeed, it underpins a further observation that a range of GelMA concentrations with consequently differing substrate stiffnesses equally supported myoblast differentiation. While the optimal stiffness for C2C12 myofibers has been reported to be ~12 kPa, it is known that actomyosin striation will occur on a second myofiber layer grown on top of a bottom myofiber layer despite a large range of underlying substrate moduli [33,34]. Our results add to this literature by demonstrating that myoblasts can further survive encapsulation in a wide range of GelMA concentrations/stiffnesses, after which the cells migrate to form a dense multilayered culture on the surface, as seen in SEM imaging. Myofibers were thus able to striate over a wide range of substrate stiffnesses due to the overlay of cells that permitted more advanced differentiation. This ability of myoblasts to compensate for the underlying modulus is an advantage for biofabrication techniques using GelMA, given that stiffer materials are generally easier to handle and have better shape fidelity when printed.

Another critical advantage of myoblast migration is that the final superficial position of the cells obviates the existing conundrum of NMJ formation with the engineered muscle. Nerve and muscle have vastly different biological characteristics that are often at odds when choosing a suitable scaffold material. In particular, neural tissues prefer much softer substrates (less than 1 kPa), which is generally incompatible with any material optimal to muscle [35,36]. Thus, the suitability of GelMA for skeletal muscle engineering may be aided by myoblast migration to the surface, which appropriately supports innervation and even vascularization. It should be noted that the fabrication of fibers with superficial cell growth is not readily achieved with the traditional approach of seeding cells on top of a pre-made scaffold, which is often hindered by poor cell migration through the matrix. Bioprinting allows for specific placement of cells throughout the entire scaffold geometry and, in the future, could incorporate fibers specific for housing regenerative muscle progenitors and delivering growth factors.

Both the molecular and functional analysis demonstrated superior myotube maturation in the bioprinted GelMA constructs when compared to the 2D controls. The gene analysis supported advanced differentiation with an inverse relationship between the expression of the two myogenic regulatory factors MYOG and MYF6, the downregulation of the early myogenic regulator SIX4, and the rapid upregulation of MYH1 and MYH8 that encode the critical contractile protein myosin. This was further reinforced with calcium imaging, which showed a progression towards organized, rhythmic calcium transients over two weeks of in vitro differentiation. This is known to represent the maturity of intracellular calcium-handling proteins that can respond to and recover from spontaneous membrane depolarization.

The bioprinted structures were then housed in chambers supplied by a surgically formed arterio-venous (AV) loop and a transected femoral nerve. This proof-of-concept study was designed to assess the feasibility of creating vascularized grafts of muscle from bioprinted structures, and whether neural outgrowth would occur in the presence of GelMA. The containment of these elements in a subcutaneous chamber enabled in vivo analysis of their interaction without confounding or synergistic effects from native muscle. The GelMA-only controls demonstrated that the chamber supported angiogenesis and neural sprouting in the presence of the material, although without any muscle formation. Immunohistochemical staining of the AV chambers after two weeks in vivo revealed bundles of regenerating as well as mature myofibers (striated and peripherally nucleated), which were integrated with neural outgrowth and neovasculature within the myoblast-containing chambers (but not in the non-myoblast-containing chambers).

Another key aspect of this experiment was the role of GelMA in vivo. GelMA transitioned from being a necessary scaffold for the initial differentiation and organization of myoblasts in vitro to a temporary support structure for in vivo myofiber differentiation and maturation. The material appeared biologically inert and was surrounded by new muscle as well as vessels and nerves. GelMA is known to degrade in vivo, giving rise to the possibility that with more time, the degradation of GelMA may yield fully tissue-engineered muscle [37,38] within the AV loop system used here. Furthermore, the very rapid differentiation and the consequential early structural independence of the developing muscle tissue frees it from being constrained by the degradation rate of GelMA or the orientation cues presented by its structural configuration.

## 4. Conclusions

The effective engineering of skeletal muscle requires a scaffold material that can deliver high cell viability, support myotube maturation and acquisition of functionality, and be permissive to innervation and vascularization. This work demonstrates myoblast migration through GelMA as a single material bioink, enabling advanced maturation and facilitating neural and vascular ingrowth. These findings provide a potential means to expedite therapeutic discovery for neuromuscular disorders by modelling fully matured, vascularized, and innervated muscle tissue in a small animal model as well as laying a potential foundation for the fabrication of innervated and vascularized muscle grafts. In future works, the application of induced pluripotent stem cells (iPSC) to generate muscle tissue constructs in the AV loop system could offer the possibility of personalized treatment options for neuromuscular diseases and volumetric muscle loss.

## 5. Materials and Methods

### 5.1. GelMA Hydrogel Synthesis

Gelatin methacryloyl (GelMA) was synthesized and sterilized, as previously described [39], with a single batch GelMA (degree of functionalization, 86%) used for this study. A stock solution of 20% *w*/*v* GelMA was made for subsequent dilutions. This was achieved by adding sterile phosphate buffered saline (PBS, Thermo Fisher Scientific) to a known mass of freeze-dried filter-sterilized GelMA, with the addition of 100 U/mL penicillin and 100 μg/mL streptomycin (Gibco). The material was dissolved at 37 °C on a shaker.

### 5.2. Photocuring

A 2% stock solution of Lithium phenyl-2,4,6-trimethylbenzoylphosphinate (LAP) (Tokyo Chemical Industry) was made with PBS, then filter-sterilized (0.22 µm filter). This concentration was used for subsequent dilutions in combination with GelMA. The material was photocured with a 365 nm UV source (Omnicure LX 400+, Lumen DynamixLDGI), without a focusing lens to allow for a more diffuse light with lower intensity. Light intensities were checked with a UV meter (Omnicure R2000 UV Radiometer) before each experiment.

### 5.3. Rheology

Rheological testing was performed on an Anton Paar Rheometer MCR 302, with a 15 mm 1° cone plate geometry and a quartz crystal stage. Temperature-dependent gelation kinetics were investigated with a temperature sweep under oscillation (1% strain and 10 rad/s) by cooling at 1.32 °C/min from 37 °C to 4 °C. Viscosity as a function of uncured GelMA was assessed as a function of shear rate (0–100/s) at 4 °C. In situ UV curing was performed with the Omnicure LX 400+ UV light source, fitted to project through the underside of the quartz crystal stage. Two UV intensities were investigated (15 mW/cm^2^ and 40 mW/cm^2^) to determine the time required for the storage modulus to plateau. The UV meter was used to measure the light intensity at the sample position.

### 5.4. Compression Testing

Different concentrations of GelMA (increments between 6–12% *w*/*v*) were prepared with 0.1% *w*/*v* LAP. Triplicate samples for mechanical testing were prepared by casting 80 µL of GelMA into molds 2 mm in depth. Samples were incubated at 4 °C for 20 min prior to crosslinking at 4 mW/cm^2^ with UV light (365 nm) at room temperature for 400 s. Samples at room temperature were similarly prepared as a point of comparison. Samples were removed from the molds and left overnight in PBS at 37 °C. Mechanical testing was performed following the protocol as previously described [40]. A TA Electro force 5500 mechanical loading device (TA Instruments, New Castle, USA) was fitted with a calibrated 5 lbf load cell. Experiments were conducted at room temperature, and samples were kept hydrated with PBS during testing. The contact area of the sample was first measured by microscopy imaging. The compression plate was lowered at 0.01 mm/s until the total displacement was 15% of the original height, which was calculated from the point of inflexion of the load vs. time curve. Load and displacement were converted into stress (σ) and strain (ε) data, respectively, using the sample surface area and height. The compressive modulus was computed using stress data between 10% and 15% strain as follows: $E_{c}=\left(\sigma_{15}-\sigma_{10}\right)/\left(\varepsilon_{15}-\varepsilon_{10}\right)$.

### 5.5. Primary Myoblast Cell Culture

Mouse myoblast cultures were prepared from skeletal muscle removed from the hind limbs of three- to four-week-old C57BL/6 mice, as previously described [41]. The muscle tissue was finely minced with scissors in a digestion buffer (Ham’s F-10 (Gibco), 400 U/mL penicillin, 400 µg/mL streptomycin, 1 µg/mL amphotericin B (Gibco), and 2.5 mM calcium chloride). An amount of 10 mg/mL Collagenase D (Roche) and 2.4 U/mL Dispase II (Roche) were added, and the tissue incubated for two hours at 37 °C. The muscle slurry was then pre-plated using plain tissue-culture flasks: twice for 20 min, once for 40 min, then at 24 h intervals for the next five days in myoblast growth media (Ham’s F-10 (Gibco), 20% fetal bovine serum (Gibco), 2.5 ng/mL recombinant human basic fibroblast growth factor (bFGF), 2 mM L-glutamine (Gibco), 100 U/mL penicillin, and 100 μg/mL of streptomycin (Gibco)). Myoblasts were maintained in growth media at 37 °C under 5% CO_2_ and passaged at 80% confluence with a dissociation buffer (8.5 mM NaCl, 0.5 mM KCl, 2.3 mM NaHCO_3_, 0.8 mM NaH_2_PO_4_.2H_2_O, 0.56 mM glucose, 0.096 mM EDTA, 10 ng/mL phenol red, and trypsin (Life Technologies) at 0.25%) and resuspended in myoblast proliferation media.

### 5.6. Myoblast Encapsulation in GelMA

Myoblasts cultured to 70–80% confluency in tissue culture flasks were trypsinized, counted, and resuspended in growth media. The cell suspension was combined with GelMA warmed to 37 °C, 0.1% LAP, 100 U/mL penicillin, and 100 µg/mL of streptomycin. The volumes of cell suspension and 20% *w*/*v* stock GelMA were titrated to form the necessary final concentrations of GelMA. For testing of GelMA for cell viability and differentiation, a concentration of five million cells/mL was used to cast into 96-well plates with a volume of 40 µL per well (average thickness of 1 mm). Ultra-low attachment wells (for suspension cultures) were used for these experiments. Photocuring was performed with exposure to 4 mW/cm^2^ UV light (365 nm) at room temperature for 400 s. After curing, the gels were gently washed with PBS, covered in myoblast growth media, and incubated in tissue culture conditions at 37 °C and 5% CO_2_. For bioprinting, the same process was repeated but with 20 million cells/mL [42]. The bioink was transferred to a sterilized printing cartridge (CELLINK) and incubated at 4 °C for 20 min.

### 5.7. Bioprinting

Bioprinting was performed using a commercial printer (INKREDIBLE+, CELLINK), conducted at room temperature. For printing, 27G cone-shaped nozzles (Nordson EFD) were used. A parametric study on fiber diameter as a function of printing speed was performed first on 8% *w*/*v* GelMA that had been cooled in the cartridge to 4 °C for 20 min prior to printing. Square crosshatch grids (two layers of 10 mm fibers laid down at 0° and 90°, spaced 2.5 mm apart, and finished with an outer 11 mm x 11 mm border) were printed in 6-well tissue culture plates at speeds between 500 and 1250 mm/min. Plates were kept at 4 °C prior to printing. Printing pressures were kept at an average of 60 kPa, which was the pressure required to initiate and maintain a steady flow of ink. Fibers were crosslinked and left in PBS overnight at 37 °C. Fiber diameters were measured in three areas in triplicate samples at each speed. The bioink formulation (with cells) was then printed at 1000 mm/min. After printing, constructs were immediately photocured with exposure to 4 mW/cm^2^ UV light (365 nm) at room temperature for 400 s. After curing, the gels were gently washed with PBS, covered in myoblast growth media, and incubated in tissue culture conditions at 37 °C and 5% CO_2_.

### 5.8. Myoblast Differentiation

After photo-crosslinking, GelMA constructs (cast or printed) were kept in myoblast growth media at 37 °C under 5% CO_2_. After 24 h, the media was changed to myoblast differentiation media (DMEM (Lonza), 2% horse serum (Gibco), 2 mM L-glutamine (Gibco), 100 U/mL penicillin, and 100 µg/mL streptomycin (Gibco)). Cells were maintained with daily half-media changes for the duration of culture. After 48 h, printed constructs were eased off the bottom of the tissue culture plate with a pipette tip if they were not already afloat in the media.

### 5.9. Myoblast Cell Viability

Myoblast density in 2D cultures was 10,000 cells/well in a 96-well plate, with a change from growth to differentiation media the day after seeding. Myoblast density for 3D cultures was five million cells/mL of GelMA, again with a change to differentiation media the day after seeding. Two cytotoxicity screening tests were performed: comparing different concentrations of LAP with adjusted UV crosslinking times, and incubation of cultures at 4 °C for 20 min before transfer to tissue culture conditions. Three time-points at day 0 (24 h after seeding), 7 and 14 of differentiation were assessed. Cell viability was measured by removing culture medium and incubating the cells with 1 µM calcein-AM (green fluorescence) and 2 µM ethidium homodimer (red fluorescence) in sterile PBS. Cells were incubated for 30 min at 37 °C, and then observed under a fluorescence microscope (EVOS XL Core cell imaging system, Thermo Fisher Scientific). Cell cytotoxicity for different concentrations of LAP was also assessed with Cell Titer-Blue tests (Promega) for the day 0 timepoint, as per the manufacturer’s protocols, and in triplicate with the fluorescent signal acquired with a CLARIOstar microplate reader (BMG LABTECH). Cell viability of myoblasts after bioprinting was also investigated at three time-points with the same techniques as above, at days 0 (24 h after printing), 7, and 14 of differentiation. The number of dead cells was counted with Image J software (National Institute of Health) in three fields at 10× magnification. The final unit for quantifying cell death was the number of dead cells per 0.1 mm^2^ of fiber area.

### 5.10. Fluorescent Staining and Imaging

GelMA-myoblast constructs were fixed with 10% formalin for 30 min, then blocked and permeabilized for an hour with 10% normal donkey serum made up with a PBS of 0.1% TritonX-100. Immunofluorescent staining was performed for sarcomeric myosin (mouse anti-MF20, Developmental Studies Hybridoma Bank). Cells were incubated in the primary antibody (1:400) overnight at 4 °C. Cells were then incubated with the secondary antibody Alexa Fluor 594-conjugated donkey anti-mouse IgG (1:2000, Molecular Probes) and Alexa Fluor 488 Phalloidin (1:100, Thermo Fisher Scientific) for 60 min at 37 °C. Nuclei were stained with 1 µg/mL of 4′,6-diamidino-2-phenylindole (DAPI, Sigma-Aldrich) for 15 min at room temperature. Samples were washed in PBS and imaged with an inverted fluorescence microscope (Olympus IX70). The 3D rendered z-stack images were taken with confocal microscopy. A total of 0.5 µm red fluorescent beads at a concentration of 25 µL/mL were added to the bioink (aqueous suspension of carboxylate–modified polystyrene latex beads, Sigma-Aldrich). After printing, the cells were then stained with Alexa Fluor 488 Phalloidin, as described above. Confocal imaging was performed with a NikonA1Plus confocal microscope using a Nikon Plan Fluor 20× DIC L N1 N.A. 0.75 objective lens, and the images were processed using NIS-Elements software (Nikon).

### 5.11. RT-qPCR

Real-time quantitative reverse transcription polymerase chain reaction (RT-qPCR) was performed on a Quant Studio 6 Flex Real-Time PCR system. Total RNA from bioprinted constructs and 2D control myoblast cultures (grown on tissue culture plastic) were harvested at Days 0, 3, 7, and 14 of differentiation with TRIzol Reagent (Ambion, Thermo Fisher Scientific). The bioprinted constructs were broken down by snap-freezing in liquid nitrogen and then ground with a mortar and pestle. The RNA was purified using the RNeasy Microkit (Qiagen) and assessed with nanodrop quantification (CLARIOStar Monochromator Microplate Reader, BMG Labtech). Reverse transcription was performed using an Omniscript RT kit (Qiagen) for 450 ng of RNA. Expression of MYOG, MYF6, SIX4, MYH1, and MYH8 was evaluated with SYBR Green Real-Time PCR Master Mix assays (Thermo Fisher Scientific). The 2^ΔΔCT^ comparative method was used to evaluate relative changes in gene expression with GAPDH as the housekeeping gene [43]. Statistical analysis was performed with unpaired t-tests on three technical replicates. The relevant primers are listed in Table 1.

### 5.12. Scanning Electron Microscopy (SEM) and Scanning Electron Cryomicroscopy (cryoSEM)

Samples were prepared for SEM as follows: bioprinted constructs were fixed in 2.5% paraformaldehyde for 30 min at 37 °C, then rinsed three times with a sodium cacodylate buffer for five minutes each. Samples were passed through successive dehydration steps in ethanol (50%, 70%, 90%, and 95% ethanol for 10 min, and 100% ethanol for 15 min). The samples were then dried by soaking in a hexamethyldisilazane (HMDS) solution overnight and attached on the SEM stubs. Finally, the samples were sputter coated with 10 nm gold. Images were observed using FEI Verios 460L FEGSEM under high vacuum conditions. Samples were prepared for cryoSEM as follows: bioprinted constructs were positioned onto a cryoSEM sample holder and plunged into liquid nitrogen (LN_2_) slush to snap freeze samples and avoid ice crystal formation. Frozen samples were placed in a sample preparation chamber maintained at −180 °C under high vacuum conditions. Next, samples were sublimated at −90 °C for two minutes. Finally, samples were gold-sputter coated for 120 s. Images were observed using FEI Quantra 200 in cryoSEM mode, at −180 °C under high vacuum. Images were taken at 15 kV.

### 5.13. Calcium Imaging

Intracellular calcium transients were assessed by loading bioprinted grids with 5 µm Fluo-4 AM dye in extracellular recording solution (145 mM NaCl, 5 mM KCl, 2.6 mM CaCl_2_, 1 mM MgCl_2_, 10 mM Na-HEPES, and 5.6 mM D-glucose at pH 7.4) [44]. After 20 min of incubation at 37 °C, the dye was removed, and then the cells were incubated for another 20 min in fresh extracellular solution. Activity was observed under a fluorescent microscope (Eclipse FN1, Nikon) and recorded with Visiview imaging software (Visitron Systems GmbH). Fluorescence images were recorded with an iXon Ultra camera at 6.67 Hz for 90 s (Oxford Instruments). Videos were analyzed with ImageJ software (National Institute of Health). Calcium transients were expressed as ΔF/F ((F_max_ − F_rest_)/F_rest_).

### 5.14. In Vivo Study

A computer-aided design (CAD) file was created using Tinkercad (Autodesk Inc.) for the 3D printing of chambers to house the bioprinted muscle and AV loop. The chamber design was based on similar devices previously described [22,45], with modifications to accommodate for the bioprinted muscle. Structures were printed in high quality, glossy-finish settings with MED610 (Stratasys) on an Objet30 3D printer (Stratasys). The chambers were cleaned and sterilized following a previously described protocol [46]. The chambers were soaked in sterile PBS overnight before use. Bioprinted grids of muscle were differentiated in vitro for one week, prior to transfer into chambers for in vivo implantation. Two chambers were prepared with bioprinted muscle, while two additional chambers were prepared with GelMA-only (acellular) grids to serve as controls. This study was approved by the St. Vincent’s Hospital Animal Ethics Committee (Melbourne, AEC Ref No: 010/18-r3) and conducted at St. Vincent’s Experimental and Medical Surgical Unit (EMSU) in accordance with the institutional ethics guidelines. Male CBH/rnu/rnu (nude rats) (ARC, Perth, Western Australia) were used for this experiment. Animals weighed a minimum of 250 g (15–16 weeks old). All animals were anaesthetized with oxygen/isoflurane inhalation (2 L O_2_/2% isoflurane) and received a subcutaneous dose of Carprofen (5 mg/kg) pre-operatively. Prior to implantation, the differentiated bioprinted muscle grids were transferred onto the base of the chamber in a class II biosafety cabinet before being taken to the sterile operating field. Samples were kept hydrated with media up until implantation. Loop surgeries were performed by animal technicians with expertise in microsurgery at EMSU, as previously described [22,23]. In brief, an AV loop was constructed in the left groin by first taking a vein graft from the right femoral vein. The vein was interposed between proximal stumps of the right femoral artery and vein with end-to-end anastomosis using 10-0 monofilament nylon sutures. This loop was placed in the chamber along with the transected femoral nerve. The chamber was secured to surrounding tissue with 6-0 prolene sutures through holes in the outer perimeter of the base. The chamber was kept open during the whole procedure to observe patency (pulsation) of the AV loop. The lid was clipped on top of the chamber, and the skin was closed in layers with 4.0 silk sutures. Animals were monitored post-operatively until they were recovered from anesthesia and were able to move around the cage. Post-operative care and monitoring were carried out as per institutional guidelines. All animals remained well throughout the two-week period with good wound healing and general health. After two weeks, the animals were anaesthetized, and the chambers wholly retrieved by resecting the neurovascular bundle at the chamber entrance. The contents of the chamber were submerged in 10% formalin for 24 h, sectioned at 3 mm intervals, and processed into paraffin wax.

### 5.15. Histology

Paraffin blocks were cut at 5 µm sections for hematoxylin and eosin (H&E) staining as well as immunohistochemistry. Immunofluorescent staining was performed for desmin (rabbit anti-desmin, 1:100, Abcam) and beta-III tubulin (mouse anti-B3T, 1:200, Covance). Sections were dewaxed in three changes of 100% xylene, rehydrated in ethanol series (100%, 80%, 60%, 30%, and water for five minutes each) and underwent antigen retrieval in a citrate buffer (10 mM, pH 6). Sections were washed and blocked in 10% goat serum for one hour at room temperature. Primary antibodies were added to the slides and left overnight at 4 °C. Cells were incubated in the secondary antibodies Alexa Fluor 594-conjugated goat anti-mouse IgG (1:2000, Molecular Probes) and Alexa Fluor 488-conjugated goat anti-rabbit IgG (1:2000, Molecular Probes) for 60 min at 37 °C. Slides were washed in PBS before mounting with Fluor shield with DAPI (Abcam). Slides were imaged with an inverted fluorescence microscope (Olympus IX70). Confocal imaging was performed with a NikonA1Plus confocal microscope using a Nikon Plan Fluor 20× DIC L N1 N.A. 0.75 objective lens and images were processed using NIS-Elements software (Nikon). All samples were sectioned and stained at three different levels.

### 5.16. Statistical Analysis

Data is presented as mean ± standard deviation (SD). GraphPad Prism 5 software (San Diego, CA, USA) was used for statistical analysis. PCR data was analyzed by performing unpaired *t*-tests with statistical significance defined as *p* < 0.05.

## Figures and Tables

**Table 1:** Primer sequences.

Name	Forward Primer	Reverse Primer
Myogenin (MYOG)	5′-GCGCCATCCAGTACATTGAGC-3′	5′-ACGATGGACGTAAGGGAGTGC-3′
Myogenic factor 6 (MYF6)	5′CGGAGTGCCATCAGCTACATTG-3′	5′-TCCACGTTTGCTCCTCCTTCC-3′
Homeobox protein SIX4 (SIX4)	5′-TTC AAG GAG AAG TCG CGC AAC-3′	5′-ACT GGG GTT GCC ATC CGA TTC-3′
Myosin heavy chain 1 (MYH1)	5′-GGCACTGTGGACTACAACATCG-3′	5′-TTT CTT TCC ACC ACC GCC ACC-3′
Myosin heavy chain 8 (MYH8)	5′-CTACCAAAGGCAAGGCCGAG-3′	5′-ATCTGCTTCAGCACTAGCGTATG-3′
Glyceraldehyde 3-phosphate dehydrogenase (GAPDH)	5′-ACAACTTTGGCATTGTGGAAGGG-3′	5′-TACTTGGCAGGTTTCTCCAGGC-3′
